# Acute Myocardial Infarction in a Young Woman: Role of Cardiac Magnetic Resonance Imaging in Establishing the Diagnosis

**DOI:** 10.7759/cureus.7526

**Published:** 2020-04-03

**Authors:** Kameel Kassab, Ralph Matar, Tareq Alyousef, Saurabh Malhotra

**Affiliations:** 1 Cardiology, John H Stroger, Jr. Hospital of Cook County, Chicago, USA

**Keywords:** acute myocardial infarction, spontaneous coronary artery dissection, minoca, cardiac mri

## Abstract

Evaluation of acute coronary syndromes (ACS) in young women presents a clinical challenge. An unremarkable coronary angiogram may not exclude ACS, as spontaneous lesion revascularization, resolved coronary spams, or spontaneous coronary dissection (SCAD) can often be missed. Cardiac magnetic resonance imaging (CMR) can provide useful information in acute myocardial infarction (MI) by confirming and sizing acute infarction and delineating the etiology when angiography is inconclusive. Here, we report a case of a 39-year-old postpartum woman with a history of hyperlipidemia who presented with a one-day history of atypical angina. On presentation, she was found to have transient ST-segment elevation in high lateral leads and elevated troponin. Coronary angiography revealed a nonobstructive lesion in the first obtuse marginal branch (OM1) distribution. The patient subsequently underwent cardiac magnetic resonance imaging (MRI) for further delineation of etiology, which confirmed acute infarction in the OM1 distribution. Diagnosis of myocardial infarction with no obstructive coronary artery disease (MINOCA) secondary to acute coronary artery dissection type 2 (SCAD-2) was made. The patient was managed conservatively with medical therapy. CMR has emerged as a front-line diagnostic imaging modality in acute MI and can provide invaluable information in the confirmation and sizing of infarction, delineating tissue characteristics, establishing the etiology of infarction, and prognostication.

## Introduction

Cardiovascular disease remains the leading cause of mortality in women in the United States. Across all age groups, women have greater mortality following acute myocardial infarction (MI) and a greater prevalence of heart failure and stroke at five years post MI [1]. Evaluation of acute coronary syndromes (ACS) in young women presents a clinical challenge with a higher prevalence of non-obstructive coronary artery disease on angiography [2]. The differential diagnosis in young women presenting with chest pain and myocardial injury is rather wide. An unremarkable coronary angiogram may not exclude ACS, as spontaneous lesion revascularization, resolved coronary vasospasm, or spontaneous coronary dissection (SCAD) can often be missed. Cardiac magnetic resonance (CMR) has the ability to outline myocardial tissue characteristics, provide imaging confirmation of acute MI, and hence the accurate diagnosis of ACS, especially when the etiology of myocardial injury remains unclear.

## Case presentation

**Figure 1:**
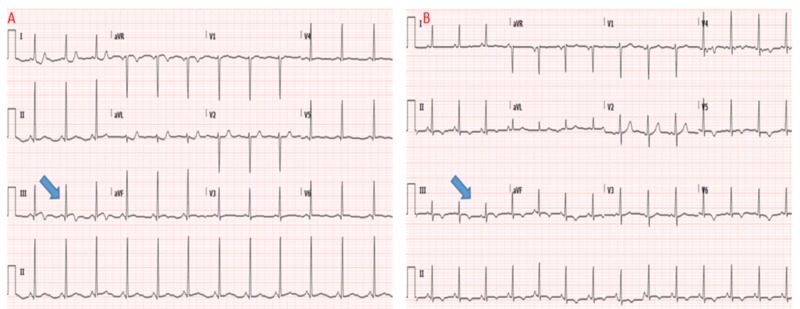
Electrocardiogram on presentation depicting ST-segment elevation in lead III with reciprocal ST-segment depression in high lateral leads (A); resolution of ECG changes coinciding with improvement of chest pain (B) ECG: electrocardiogram

A 39-year-old Hispanic woman presented to the emergency room (ER) with a one-day history of tearing substernal chest pain. The first chest pain episode occurred a day prior to presentation while she was walking and lasted 15 minutes before partially subsiding. The next day, she experienced a similar episode of exertional chest pain radiating to her back, which persisted, necessitating an ER visit. Her medical history was significant for hypertension and a history of pre-eclampsia during her fourth pregnancy seven months prior to her current presentation. She has no family history of premature coronary artery disease (CAD) or dyslipidemia. In the ER, her blood pressure was found to be elevated at 190/95 mmHg in bilateral upper extremities, and her heart rate was 90 bpm. The remainder of the physical examination was unremarkable. Electrocardiogram (ECG) at presentation revealed transient 0.5 mm ST-segment elevation in inferior leads with reciprocal ST-segment depression in high lateral leads, which resolved on repeat ECG 15 minutes later after the administration of sublingual nitroglycerine and improvement of her chest pain (Figure 1).

**Figure 2:**
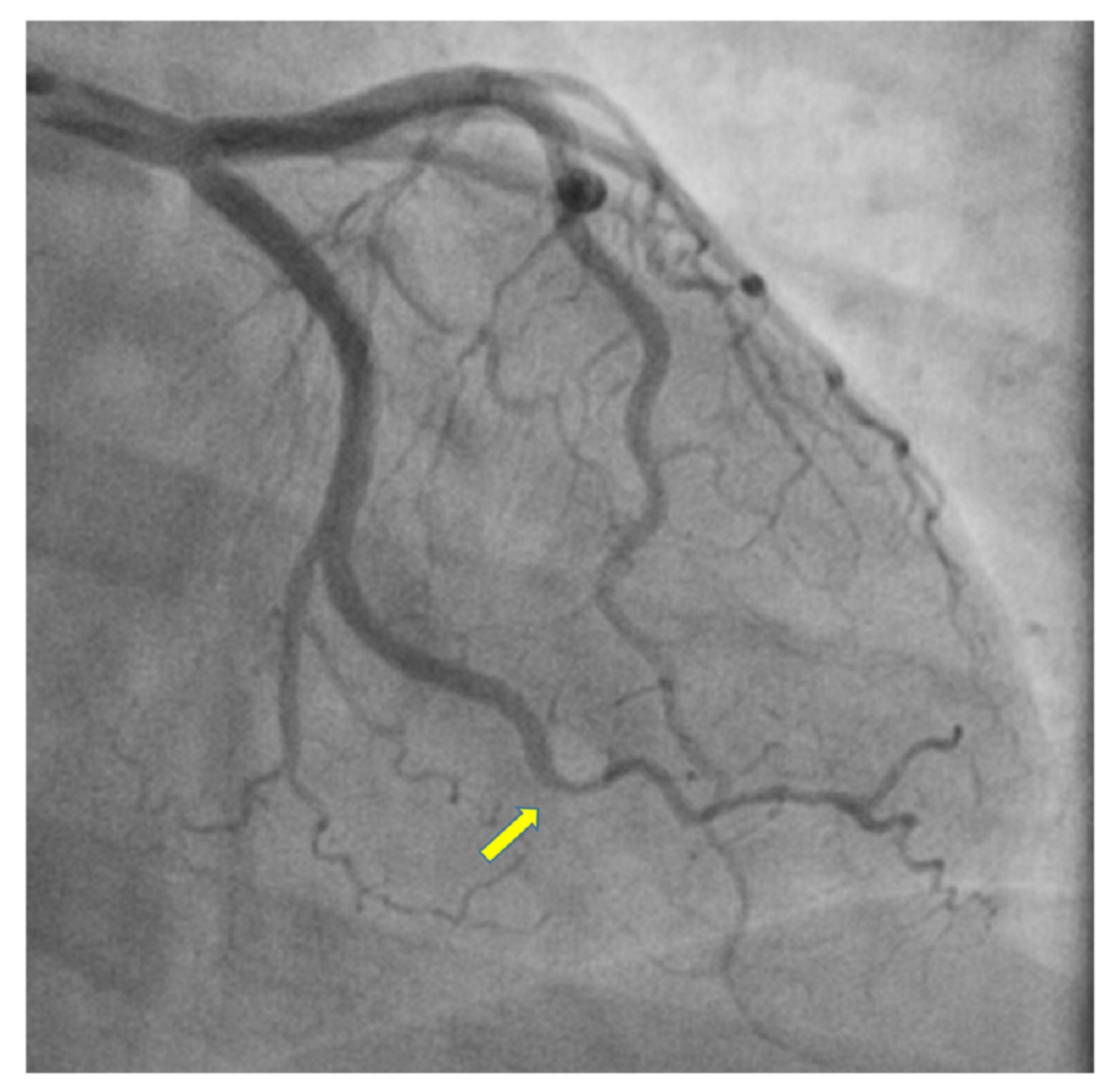
Right anterior oblique caudal view on coronary angiography revealing obtuse marginal 1 branch lesion suggestive of type 2 SCAD SCAD: spontaneous coronary dissection

Troponin I on presentation was 0.7 ng/ml and subsequently up-trended to 2.4 ng/ml three hours later. CT angiography of the chest, abdomen, and pelvis did not show any evidence of aortic dissection or pulmonary embolism. She was loaded with 325 mg of aspirin and started on intravenous (IV) heparin drip infusion in the ER and admitted to the cardiac intensive care unit for the further management of acute MI. Her third troponin set up-trended to 20 ng/ml. She was taken for invasive coronary angiography early the next morning. Coronary angiogram revealed right-sided dominant circulation with diffuse narrowing in the distal segment of the first obtuse marginal (OM1) (Figure 2, Video 1).

**Video 1:** Coronary angiogram depicting left coronary circulation in LAO cranial (Panel A), RAO cranial (Panel B), and RAO caudal (Panel C) projections. Right coronary circulation is depicted in LAO projection (Panel D) LAO: left anterior oblique; RAO: right anterior oblique

**Figure 3:**
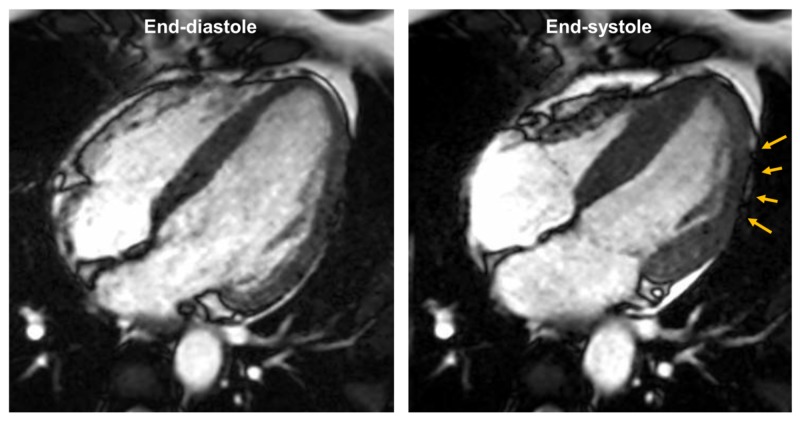
Cine cardiac magnetic resonance in four-chamber orientation There is akinesis of the mid to apical inferolateral wall, with a lack of myocardial thickening (orange arrows) in this region as evidenced from a comparison of the end-diastolic and end-systolic frames.

No other coronary lesion could be identified. Due to the inability of the coronary angiogram to provide a definitive diagnosis, the patient was referred for cardiac magnetic resonance imaging (CMR) to provide imaging confirmation of infarction and further delineate the etiology. Contrast-enhanced CMR was performed on a 1.5 Tesla Toshiba Vantage Titan system (Canon Medical Systems Corporation, Tochigi, Japan) after the administration of 40 ml of gadolinium-based contrast (Omniscan, GE Healthcare, Chicago, Illinois). Cine CMR images revealed akinesis of the mid to apical inferolateral wall (Figure 3).

**Figure 4:**
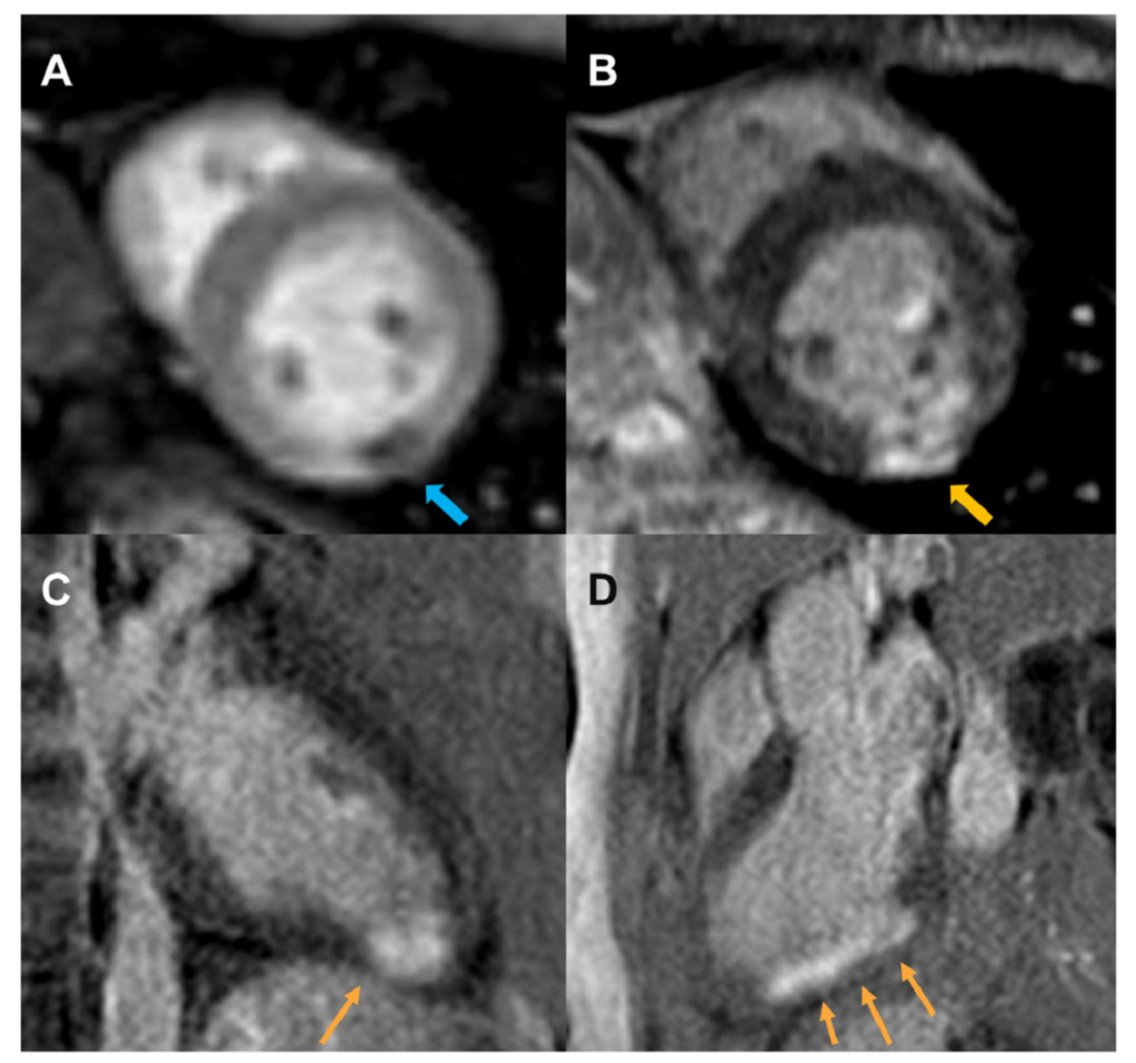
Gadolinium-enhanced cardiac magnetic resonance First-pass perfusion image depicting a focal region of transmural hypoperfusion (blue arrow) in the mid inferolateral segment of the left ventricle in a short-axis orientation (Panel A). Delayed contrast-enhanced sequences depicting a transmural scar in the inferior wall on the short-axis view (Panel B; inversion recovery sequence) and in the apical inferior wall in the two-chamber view (Panel C; phase-sensitive inversion recovery sequence). Transmural infarction of the inferolateral wall is noted in the three-chamber view (Panel D; phase-sensitive inversion recovery sequence).

First-pass perfusion images showed focal transmural hypoperfusion of the mid inferolateral segment (Figure 4A), with transmural myocardial hyperenhancement of the mid to apical inferolateral wall (Figures 4B-4D).

The rest of her workup was notable for low-density lipoprotein (LDL) cholesterol of 203 mg/dl. A subsequent hospital course was unremarkable and she was discharged on aspirin, beta-blockers, angiotensin-converting enzyme inhibitors (ACEI), and high-intensity statin. Based on the findings of contrast-enhanced CMR that were consistent with a subendocardial infarction in the distribution of the left circumflex artery and angiographic evidence of diffuse narrowing of the OM1, a presumptive diagnosis of type 2 spontaneous coronary artery dissection was made.

## Discussion

We describe a case of a young woman, seven-month post-partum after her fourth pregnancy, who is presenting with acute MI as defined by the fourth universal definition of myocardial infarction [3]. Evaluation of myocardial injury in young women could present a significant clinical challenge since obstructive atherosclerotic disease is less likely, yet still possible, especially in patients with certain risk factors, such as dyslipidemia, like in our patient. Alternative coronary pathologies, including coronary artery spasm (CAS) and SCAD, would be as likely. SCAD represents only 1%-4% of all ACS causes yet is the cause of acute MI in up to 35% of women younger than 50 years of age [4]. Pregnancy-associated SCAD has been reported as early as five weeks of gestation to as late as a year post-partum especially in lactating women [4]. Our patient did fit into the demographics of SCAD given her age, sex, multiparty, post-partum status, and lack of traditional atherosclerotic risk factors other than dyslipidemia. Coronary angiography is the gold standard for the evaluation of coronary anatomy in patients presenting with ACS and in our patient, it did depict a diffuse lesion in the terminal portion of the OM1 branch, raising suspicion for type 2 acute coronary dissection (characterized by luminal compression without evidence of a dissection flap). However, as many lesions would spontaneously re-vascularize by the time of angiography, it is possible for the coronary angiogram not to provide a definitive diagnosis.

Commonly, intracoronary imaging with intravascular ultrasound or optical coherence tomography is instrumental in confirming the diagnosis in subtle cases where lesion appearance is unclear such as type 3 SCAD [4-5]. However, their use is frequently avoided due to concern for extending the coronary dissection with wire or imaging catheter, inducing guide-catheter iatrogenic dissection or causing catheter-induced occlusion of the true lumen [4-5]. In our patient, the lesion had a characteristic angiographic appearance of type 2 SCAD, obviating the absolute need for intravascular imaging. CAS is usually a cause of recurrent angina associated with transient ST elevation and is thought to be mediated by vagal withdrawal, vascular smooth muscle hyperactivity, and an imbalance of the autonomic nervous system. Smoking is a major predisposing risk factor. Women presenting with CAS are usually older and have less significant obstructive CAD [1]. In our patient, the initial resolution of chest pain with the administration of nitroglycerin in the ER could indicate improvement of vasospasm, which could be subsequently associated with an unremarkable coronary angiogram. Yet, some degree of atherosclerosis is usually expected on an angiogram, which was not the case in our patient. In addition, nitroglycerin was repeatedly administered during coronary angiography without any significant effect on the angiographic appearance of the lesion. Plaque disruption which encompasses plaque erosion, rupture, and calcific nodules can cause infarction via thrombus formation with distal embolization, superimposed arterial spasm, or complete thrombosis [6]. Plaque disruption could be suggested via angiographic appearance yet definitive diagnosis can only be achieved via intracoronary imaging. In our patient, angiography was not suggestive of recent plaque disruption. In situ coronary thrombosis or embolic phenomena to the coronary circulation were also less likely. Myocardial infarction in the absence of obstructive coronary artery disease (MINOCA) compromises 6% of acute MI, carries a similar prognosis to type 1 MI and remains a diagnosis of exclusion. Women presenting with acute MI are more than twice as likely as men to have MINOCA [6]. It encompasses patients with evidence of atherosclerosis that is not considered sufficiently severe to compromise myocardial blood flow [6]. Hence, the absence of obstructive coronary artery disease on an angiogram does not exclude acute MI. Various etiologies, including plaque disruption, coronary thrombosis, vasospasm, coronary dissection, and microvascular dysfunction, have been implicated in MINOCA [3,6].

Whether our patient did have a true infarct remained unclear until it was confirmed by CMR. It has an advantage over other imaging modalities, as it allows for the characterization of myocardial substrate both with non-contrast and contrast-enhanced sequences. CMR has been validated in the detection of ACS, where it has been suggested that it may identify ACS more accurately than conventional markers [7]. In a study of 1345 patients with diagnosed STEMI, 127 (9.5%) were found on angiography to have no CAD. CMR in these patients had a diagnostic yield of 75%, differentiating 31% as myocarditis, 31% as stress cardiomyopathy, and 29% as STEMI without an angiographic lesion [8]. In several other cohorts of patients presenting with chest pain, elevated troponins, and non-obstructive CAD on angiography, CMR persistently showed a high diagnostic yield in identifying the infarction area and delineating the etiology of infarction [9-10]. CMR also provides important prognostic implications in patients post ACS, as it allows for the accurate assessment of infarct size and the peri-infarct zone, which are independent predictors of post-MI mortality [11]. In addition, the presence of late gadolinium enhancement is a powerful predictor of ventricular arrhythmic risk in patients with ventricular dysfunction [12]. CMR ruled out myocarditis and stress cardiomyopathy and showed a discrete infarct in the OM1 distribution with corresponding regional wall motion abnormalities. Combining angiographic and CMR data, MINOCA secondary to type 2 SCAD would be the probable diagnosis in our case.

## Conclusions

Mortality burden post MI remains disproportionately higher in women, making the early recognition, diagnosis, and treatment of paramount importance. The differential diagnosis of acute MI presentation in young women remains wide, creating a diagnostic and prognostic challenge. CMR has emerged as a feasible and reliable tool in the early diagnosis and management of acute coronary syndromes. CMR confers a high diagnostic yield and aids in detection and prognostication following MI.

## References

[1] Mehta LS, Beckie TM, DeVon HA (2016). Acute myocardial infarction in women. A scientific statement from the American Heart Association. Circulation.

[2] Garcia M, Mulvagh SL, Merz CN, Buring JE, Manson JE (2016). Cardiovascular disease in women: clinical perspectives. Circ Res.

[3] Thygesen K, Alpert JS, Jaffe AS (2018). Fourth universal definition of myocardial infarction (2018). Circulation.

[4] Hayes SN, Kim ESH, Saw J (2018). Spontaneous coronary artery dissection: current state of the science: a scientific statement from the American Heart Association. Circulation.

[5] Saw J, Mancini GBJ, Humphries KH (2016). Contemporary review on spontaneous coronary artery dissection. J Am Coll Cardiol.

[6] Tamis-Holland JE, Jneid H, Reynolds HR (2019). Contemporary diagnosis and management of patients with myocardial infarction in the absence of obstructive coronary artery disease: a scientific statement from the American Heart Association. Circulation.

[7] Kwong RY, Schussheim AE, Rekhraj S (2003). Detecting acute coronary syndrome in the emergency department with cardiac magnetic resonance imaging. Circulation.

[8] Larson DM, Menssen KM, Sharkey SW (2007). "False-positive" cardiac catheterization laboratory activation among patients with suspected ST-segment elevation myocardial infarction. JAMA.

[9] Assomull RG, Lyne JC, Keenan N (2007). The role of cardiovascular magnetic resonance in patients presenting with chest pain, raised troponin, and unobstructed coronary arteries. Eur Heart J.

[10] Bhatia S, Anstine C, Jaffe AS (2019). Cardiac magnetic resonance in patients with elevated troponin and normal coronary angiography. Heart.

[11] Lockie T, Nagel E, Redwood S, Plein S (2009). Use of cardiovascular magnetic resonance imaging in acute coronary syndromes. Circulation.

[12] Disertori M, Gonzini L, Rigoni M (2016). Myocardial fibrosis assessment by LGE is a powerful predictor of ventricular tachyarrhythmias in ischemic and nonischemic LV dysfunction: a meta-analysis. JACC Cardiovasc Imaging.

